# Cardiovascular adverse events associated with denosumab versus zoledronic acid in patients with breast cancer: a propensity score overlap weighted analysis

**DOI:** 10.1007/s10549-025-07852-x

**Published:** 2025-11-25

**Authors:** Chikako Iwai, Takaaki Konishi, Atsushi Miyawaki, Akira Okada, Toshiaki Isogai, Taisuke Jo, Hideo Yasunaga

**Affiliations:** 1 Department of Clinical Epidemiology and Health Economics, School of Public Health, The University of Tokyo, 7-3-1 Hongo, Bunkyo-ku, Tokyo, 113-0033 Japan; 2 Department of Breast and Endocrine Surgery, Graduate School of Medicine, The University of Tokyo, Tokyo, Japan; 3 Division of Health Services Research, National Cancer Center Institute for Cancer Control, Tokyo, Japan; 4 Department of Health Services Research, Graduate School of Medicine, The University of Tokyo, Tokyo, Japan; 5 Public Health Research Group, Institute of Medicine, University of Tsukuba, Ibaraki, Japan; 6 Department of Prevention of Diabetes and Lifestyle-Related Diseases, Graduate School of Medicine, The University of Tokyo, Tokyo, Japan; 7 Department of Cardiology, Tokyo Metropolitan Tama Medical Center, Tokyo, Japan

**Keywords:** Bone resorption inhibitors, Breast cancer bone metastasis, Cardiovascular events, Fracture, Mortality

## Abstract

**Background:**

To compare the risks of cardiovascular events, fractures, and all-cause mortality between denosumab and zoledronic acid in patients with breast cancer bone metastases.

**Patients and methods:**

We identified female patients with breast cancer and bone metastases who received denosumab or zoledronic acid between April 2014 and August 2023 from a nationwide database of routinely collected administrative claims data in Japan. After adjusting for potential confounders using propensity score overlap weighting, we estimated the incidence of outcomes (per 10,000 person-years) and hazard ratios (HRs) using Cox proportional hazards models.

**Results:**

Among the eligible 4350 patients, 2953 received denosumab and 1397 received zoledronic acid. The participants’ median age was 76 years (interquartile range, 68 to 81). The adjusted incidence of composite cardiovascular disease was 118 in the denosumab group and 152 in the zoledronic acid group (HR 0.80, 95% confidence interval, 0.67 to 0.95). Heart failure was less frequent in patients administered denosumab [65 vs. 92; HR, 0.69 (0.55 to 0.87)] than in those administered zoledronic acid, whereas the rates of stroke and myocardial infarction were similar between the two groups. Denosumab was also associated with lower risks of any fracture [237 vs. 298; HR 0.80 (0.71 to 0.90)], hip (31 vs. 43), vertebral (135 vs. 168), and non-vertebral (114 vs. 142) fractures. Overall, 471 all-cause mortality events occurred in the denosumab group and 610 in the zoledronic acid group [HR 0.75 (0.69 to 0.82)].

**Conclusion:**

In patients with breast cancer bone metastases, denosumab was associated with lower risks of cardiovascular events, fractures, and mortality than those with zoledronic acid.

**Supplementary Information:**

The online version contains supplementary material available at 10.1007/s10549-025-07852-x.

## Introduction

Cancer survivors and patients undergoing chemotherapy face an elevated risk of developing cardiovascular disease [1–3]. A previous population-based study of over 3.2 million cancer survivors in the USA reported a nearly four-fold higher risk of cardiovascular disease mortality in the first year after cancer diagnosis compared with the general population [3]. This association is particularly marked in patients with breast cancer because specific anticancer agents (e.g., anthracycline, human epidermal growth factor receptor 2 inhibitors) can increase the risk of cardiovascular diseases [4].

Bone metastasis among patients with cancer constitutes another critical issue because skeletal-related events (SREs) greatly mar their quality of life [5]. Since breast cancer has a high frequency of bone metastasis, SREs are a highly relevant concern in this patient population [5, 6]. Bone resorption inhibitors such as the receptor activator of the nuclear factor kappa-β ligand (RANKL) inhibitor (e.g., denosumab) and intravenous bisphosphonate (e.g., zoledronic acid) are widely used to avert these debilitating complications. These agents have been shown to reduce SREs, including fractures [7, 8] and improve the quality of life of patients with bone metastases [9, 10]. A randomized trial comparing the efficacy of denosumab (a fully human monoclonal antibody against RANKL) with that of zoledronic acid for delaying or preventing SREs in patients with breast cancer bone metastases demonstrated that denosumab was superior to zoledronic acid in delaying the time to the first SRE during the study period [7].

However, evidence demonstrating whether denosumab or zoledronic acid is associated with a higher risk of cardiovascular events in patients with breast cancer bone metastases is scarce. A meta-analysis of patients with osteoporosis showed that denosumab (60 mg, administered subcutaneously every 6 months) was associated with a significantly lower risk of composite cardiovascular disease compared to zoledronic acid (5 mg, administered intravenously yearly) in patients aged 50 years and above [11]. The doses of denosumab (120 mg, administered subcutaneously every 4 weeks) and zoledronic acid (3–4 mg, intravenously every 3–4 weeks) for bone metastasis are higher doses than their respective doses for osteoporosis. Two randomized controlled trials in patients with breast cancer showed no significant difference in the risk of cardiovascular disease between denosumab and a placebo [12, 13]. No randomized controlled trial or observational study has compared the cardiovascular disease risk between denosumab and zoledronic acid in metastatic breast cancer [14]. Because patients with cancer already face a higher risk of cardiovascular disease [1, 2], the cardiovascular risks associated with bone resorption inhibitors may differ from those in osteoporosis treatment, which requires a lower dosage.

To address this knowledge gap, we aimed to compare the risks of cardiovascular events, fractures, and all-cause mortality between denosumab and zoledronic acid in patients with bone metastases from breast cancer.

## Methods

### Data source

This retrospective, cohort study was conducted using data extracted from the DeSC administrative claims database (DeSC Healthcare Inc. Tokyo, Japan). This database comprises claims data for 12,489,814 insurance beneficiaries covered by several Japanese public health insurers [15, 16], including: (ⅰ) employee health insurance (n = 1,133,026), (ⅱ) non-employee health insurance (n = 7,446,869), and (ⅲ) late elderly healthcare system for individuals aged ≥ 75 years (n = 3,909,919). It should be noted that mortality data were only available from non-employee health insurance and late elderly healthcare system. This reflects the differences in mortality data collection systems across insurance schemes, as the employee health insurance system collects limited mortality data by design.

Diagnoses were recorded using the *International Classification of Diseases, 10th revision* (ICD-10), and nationally standardized Japanese diagnostic codes. The drug specifications were documented using the *Anatomical Therapeutic Chemical classification* system established by the World Health Organization. Information on each prescribed drug, including date, dosage, and duration, was available. Additionally, medical procedures were documented using Japanese medical procedural codes. A validation study showed the DeSC database possesses good accuracy for certain diagnoses and procedural information [15].

The need for informed consent was waived because the patient database was anonymized. This study was approved by the Institutional Review Board of the University of Tokyo (approval number: 2021010NI, April 23, 2021).

### Patient selection

We identified female patients with breast cancer (ICD-10 code: C50) aged ≥ 18 years in whom denosumab or zoledronic acid was initiated for bone metastases between April 2014 and August 2023. The initiation date of either drug was defined as the index date. The exclusion criteria were as follows: (i) missing mortality data; (ii) patients who joined the insurers included in the database within 1 year preceding the index date (i.e., those without a washout period); (ⅲ) intravenous zoledronic acid administration (5 mg annually) within the washout period (because the patient was deemed to have osteoporosis already); and (ⅳ) subcutaneous denosumab administration (60 mg every 6 months) within the washout period (because the patient was deemed to have osteoporosis already). Eligible patients were divided into the denosumab (120 mg every 4 weeks, subcutaneously) and zoledronic acid (3–4 mg every 3–4 weeks, intravenously) groups. The study design is depicted in Fig. S1.

### Outcomes

The primary outcome was the first occurrence of a composite cardiovascular event requiring hospitalization. This composite included heart failure, myocardial infarction, and stroke. Heart failure was defined as hospitalization under a diagnosis of heart failure and the initiation of intravenous furosemide, carperitide, or tolvaptan [17, 18] within 30 days of admission.

The secondary outcomes included the individual components of the primary cardiovascular outcome as well as a composite outcome of any fracture (hip, vertebral, or non-vertebral), each type of fracture event, and all-cause mortality. The composite outcome of any fracture included hip, vertebral, and nonvertebral fractures. Non-vertebral fractures included fractures of the pelvis, femur, leg, ankle, shoulder, forearm, and wrist.

Follow-up was initiated on the date of the first administration of bone resorption inhibitors and was censored at the incidence of the outcomes, exit from the database, or August 2023, whichever occurred first. Disease names were identified based on the ICD-10 codes (Table S1).

### Adjustment variables

The covariates included patient background (age, comorbidities, and medications), breast cancer treatment (surgery, radiotherapy, hormonal agents, and chemotherapy), hospital type, region, and treatment years.

Age at the index date was categorized into ten groups: 18–39, 40–44, 45–49, 50–54, 55–59, 60–64, 65–69, 70–74, 75–79, and ≥ 80 years. We investigated comorbidities such as angina, atrial fibrillation, autoimmune disease, brain metastasis, chronic renal disease, diabetes, disorders of calcium metabolism, heart failure, hypertension, myocardial infarction, osteoporosis, and stroke using the relevant ICD-10 codes (Table S1). Heart failure, myocardial infarction, and stroke were defined as events requiring hospitalization. We also calculated the Charlson Comorbidity Index [19] and categorized the values into three groups, namely, 2, 3–4, and ≥ 5. Medications included anticoagulants, antidiabetics, antiplatelets, antipsychotics, proton pump inhibitors, statins, corticosteroids, osteoporosis medications [20] (i.e., calcitonin, estrogen preparations, oral bisphosphonates, romosozumab, selective estrogen receptor modulators, and teriparatide) and intravenous bisphosphonates (ibandronic acid and pamidronic acid) for bone metastases or malignancy-associated hypercalcemia.

Given their distinct clinical contexts and risk profiles, surgery for breast cancer (e.g., mastectomy or breast-conserving surgery) and surgery for fracture treatment (e.g., fixation of femoral or vertebral fractures) were adjusted for separately in the covariate adjustment and outcome analyses. The following breast cancer pharmacotherapies with potential cardiotoxicity were also included: hormonal agents (anti-estrogen agents and aromatase inhibitors) and chemotherapy (anthracyclines, cyclin-dependent kinase 4/6 inhibitors, human epidermal growth factor receptor 2 inhibitors, immune checkpoint inhibitors, and taxanes) [4].

The hospital category included academic hospitals and designated cancer care hospitals. The geographical region was categorized into nine groups: Hokkaido/Tohoku, South Kanto, North Kanto/Koshin, Hokuriku, Tōkai, Kinki, Chugoku, Shikoku, and Kyushu/Okinawa.

Information on radiation therapy, surgery, comorbidities, and medication was obtained from the diagnostic record on the index date or within 6 months preceding the index date.

### Statistical analysis

We used the propensity-score overlap weighting method to balance the covariates between the two groups. Overlap weighting analysis minimizes the asymptotic variance of the nonparametric estimates of the weighted average treatment effect within each weight class [21–24]. We calculated the propensity scores using multivariable logistic regression analysis with all the above-mentioned covariates. We computed the weights as the probability of each patient receiving the opposite treatment. Standardized differences were calculated to assess the balance of covariates between the two groups; an absolute standardized difference ≥ 10% was considered to indicate a meaningful imbalance between the two groups [25]. Overlap weighting mathematically ensures perfect balance, reducing all standardized differences to zero [23]. We compared the Kaplan–Meier curves between the two groups using the log-rank test and calculated the incidence of outcomes ( per 10,000 person-years) and the risk difference after overlap weighting. Additionally, we estimated the hazard ratios (HRs) to assess the association between denosumab use and the outcomes using cause-specific Cox proportional hazard models in overlap-weighted cohorts. Robust variance was used to calculate the confidence intervals to account for sample weights [24, 26]. We used cause-specific Cox proportional hazard models to estimate instantaneous risks among patients who remained alive and event-free.

### Secondary analyses

We conducted several sensitivity analyses to assess the robustness of our findings.

First, we conducted a competing risk analysis, considering death as a competing risk for the sensitivity analysis [27]. We estimated subdistribution hazard ratios to assess the associations between denosumab use and outcomes using the Fine-Gray model in the overlap-weighted cohorts [28]. This analysis was prespecified as secondary, complementing the primary cause-specific Cox models.

Second, we conducted a sensitivity analysis with the full cohort, irrespective of the availability of mortality data. The main analysis included only cohorts with mortality data and excluded the employee health insurance cohort, which included many patients aged < 65 years. Sensitivity analysis including the full cohort was necessary to assess the robustness and generalizability of the findings. The follow-up period was defined as the time until the last observed data point or the end of the study.

Third, we conducted a falsification test using an outcome that is not plausibly associated with the exposure [29]. We examined the incidence of burn injuries (defined by ICD-10 codes T20–T32) as an alternative outcome. Since burn injuries are unrelated to the mechanism of action of bone resorption inhibitors, we hypothesized that their incidence would be similar between denosumab and zoledronic acid if unobserved confounding was minimal.

All hypothesis tests involved a two-sided statistical significance level of 0.05. All statistical analyses were performed using Stata/SE 19.5 statistical software (StataCorp, College Station, TX, USA).

## Results

**Fig. 1:**
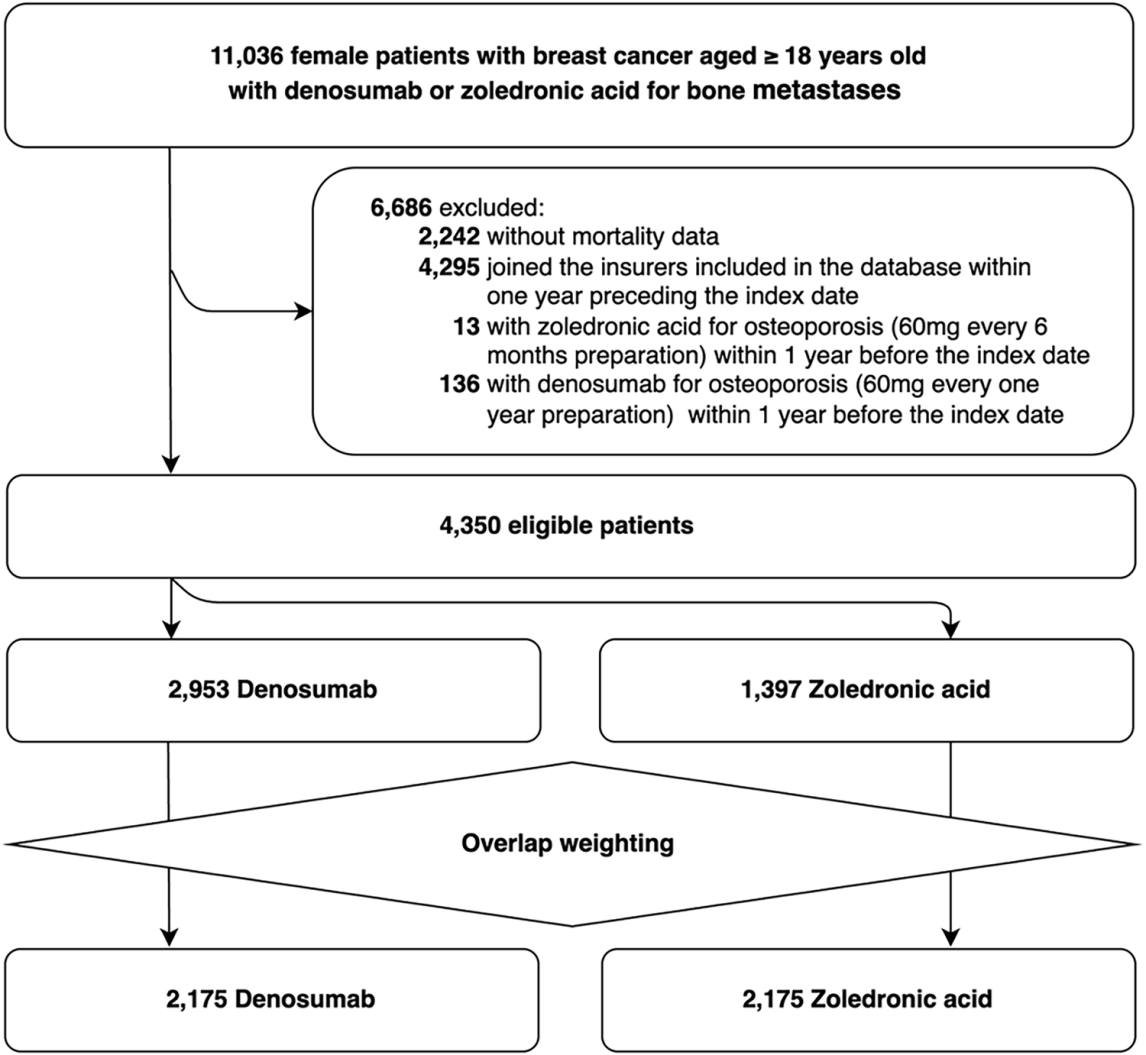
Flow diagram

We identified 11,036 female patients with breast cancer aged **≥** 18 years in whom denosumab or zoledronic acid administration was initiated. We excluded 6,686 patients based on the exclusion criteria (Fig. 1). Of the 4,350 eligible patients, the denosumab and zoledronic groups comprised 2,953 and 1,397 patients, respectively.

Tables 1 and 2 present the patients’ baseline characteristics and medications before and after propensity score overlap weighting for patients treated with denosumab or zoledronic acid. Before adjusting with overlap weighting, the Charlson Comorbidity Index and medications differed significantly between the denosumab and zoledronic acid groups. After weighting, each group comprised 2,175 patients, and the backgrounds were completely balanced (i.e., all absolute standardized differences equaled zero). Patients aged ≥ 70 years accounted for 69% of the study population. Their median age was 76 (interquartile range, 68 to 81) years. The median follow-up period was 330 (range, 129 to 704) days in the denosumab group and 224 (range, 75 to 601) days in the zoledronic acid group.

**Table 1:** Patients’ background data before and after weighting

	Before weighting					After weighting				
	Denosumab		Zoledronic acid		ASD*	Denosumab		Zoledronic acid		ASD*
	*n* = 2953		*n* = 1397		(%)	*n* = 2175		*n* = 2175		(%)
*Age category, years*										
< 40	20	(0.7)	7	(0.5)	2.3	13	(0.6)	13	(0.6)	0.0
40–44	23	(0.8)	18	(1.3)	5.0	24	(1.1)	24	(1.1)	0.0
45–49	59	(2.0)	35	(2.5)	3.4	49	(2.3)	49	(2.3)	0.0
50–54	87	(2.9)	43	(3.3)	2.0	67	(3.1)	67	(3.1)	0.0
55–59	100	(3.4)	46	(7.4)	18.0	76	(3.5)	76	(3.5)	0.0
60–64	208	(7.0)	104	(7.4)	1.5	159	(7.3)	159	(7.3)	0.0
65–69	425	(14)	179	(13)	4.6	292	(13)	292	(13)	0.0
70–7	529	(18)	230	(16)	3.8	364	(17)	364	(17)	0.0
75–79	622	(21)	253	(18)	7.4	418	(19)	418	(19)	0.0
≥ 80	880	(30)	482	(35)	10.1	713	(33)	713	(33)	0.0
*Comorbidities*										
Angina	116	(3.9)	58	(4.2)	1.1	88	(4.0)	88	(4.0)	0.0
Atrial fibrillation	63	(2.1)	26	(1.9)	1.9	42	(1.9)	42	(1.9)	0.0
Autoimmune diseases	59	(2.0)	28	(2.0)	0.0	42	(1.9)	42	(1.9)	0.0
Brain metastasis	57	(1.9)	31	(2.2)	2.0	49	(2.3)	49	(2.3)	0.0
Chronic kidney disease	40	(1.4)	25	(1.8)	3.5	29	(1.3)	29	(1.3)	0.0
Diabetes	125	(4.2)	50	(3.6)	3.4	83	(3.8)	83	(3.8)	0.0
Disorders of calcium metabolism	36	(1.2)	16	(1.1)	0.7	26	(1.2)	26	(1.2)	0.0
Fracture	113	(3.8)	62	(4.4)	3.1	92	(4.2)	92	(4.2)	0.0
Heart failure^†^	54	(1.8)	33	(2.4)	3.7	47	(2.2)	47	(2.2)	0.0
Hypertension	636	(22)	307	(22)	1.1	479	(22)	479	(22)	0.0
Myocardial infarction^†^	6	(0.2)	1	(0.1)	3.6	2	(0.1)	2	(0.1)	0.0
Osteoporosis	385	(13)	183	(13)	0.2	284	(13)	284	(13)	0.0
Stroke^†^	16	(0.5)	8	(0.6)	0.4	12	(0.6)	12	(0.6)	0.0
*Charlson comorbidity index*										
2	1,521	(52)	664	(48)	8.0	1,064	(49)	1,064	(49)	0.0
3–4	328	(11)	160	(11)	1.1	242	(11)	242	(11)	0.0
≥ 5	1,104	(37)	573	(41)	7.4	869	(40)	869	(40)	0.0
*Medications*										
Anticoagulants	196	(6.6)	93	(6.7)	0.1	148	(6.8)	148	(6.8)	0.0
Antidiabetics	452	(15)	192	(14)	4.4	308	(14)	308	(14)	0.0
Antiplatelets	404	(14)	190	(14)	0.2	294	(14)	294	(14)	0.0
Antipsychotics	775	(26)	458	(33)	14.4	664	(31)	664	(31)	0.0
Corticosteroids	1,129	(38)	711	(51)	25.7	438	(20)	438	(20)	0.0
Disorders of calcium metabolism	36	(1.2)	16	(1.1)	0.7	26	(1.2)	26	(1.2)	0.0
Intravenous bisphosphonates^‡^	53	(1.8)	32	(5.9)	21.6	44	(2.0)	44	(2.0)	0.0
Proton pump inhibitors	1,296	(44)	706	(51)	13.3	1,061	(49)	1,061	(49)	0.0
Statins	840	(28)	347	(25)	8.2	568	(26)	568	(26)	0.0
*Osteoporosis agents*										
Calcitonin	68	(2.3)	118	(8.4)	27.5	101	(4.6)	101	(4.6)	0.0
Estrogen preparations	24	(0.8)	14	(2.3)	12.0	18	(0.8)	18	(0.8)	0.0
Oral bisphosphonates	209	(7.1)	83	(5.9)	4.6	136	(6.3)	136	(6.3)	0.0
Romosozumab	16	(0.5)	4	(0.3)	4.0	7	(0.3)	7	(0.3)	0.0
Selective estrogen receptor modulators	94	(3.2)	41	(2.9)	1.4	69	(3.2)	69	(3.2)	0.0
Teriparatide	28	(0.9)	20	(1.4)	4.5	26	(1.2)	26	(1.2)	0.0

The values represent numbers (percentages), unless stated otherwise

*ASD* Absolute standardized difference

*An ASD ≥ 10% was considered to indicate a meaningful imbalance between the two groups

^†^Heart failure, myocardial infarction, and stroke were defined as events that occurred during hospitalization

^‡^Intravenous bisphosphonates included ibandronic acid and pamidronic acid

**Table 2:** Treatment background before and after weighting

	Before weighting					After weighting				
	Denosumab		Zoledronic acid		ASD*	Denosumab		Zoledronic acid		ASD^*^
	*n* = 2953		*n* = 1397		(%)	*n* = 2175		*n* = 2175		(%)
Radiation therapy	794	(27)	343	(25)	5.3	567	(26)	567	(26)	0.0
Surgery for breast cancer	40	(1.4)	22	(1.6)	1.8	22	(1.0)	22	(1.0)	0.0
Surgery for facture	13	(0.4)	10	(0.7)	3.6	14	(0.6)	14	(0.6)	0.0
*Hormonal agents*										
Anti-estrogen agents	654	(22)	181	(13)	24.3	334	(15)	334	(15)	0.0
Aromatase inhibitors	914	(31)	245	(18)	31.7	457	(21)	457	(21)	0.0
*Chemotherapy*										
Anthracyclines	71	(2.4)	50	(3.6)	6.9	70	(3.2)	70	(3.2)	0.0
Cyclin-dependent kinase 4/6 inhibitors	401	(14)	72	(5.2)	29.2	147	(6.8)	147	(6.8)	0.0
HER2 inhibitors	115	(3.9)	111	(7.9)	17.2	140	(6.4)	140	(6.4)	0.0
Immune checkpoint inhibitors	106	(3.6)	49	(3.5)	0.4	82	(3.8)	82	(3.8)	0.0
Taxane	299	(10)	196	(14)	12.0	288	(13)	288	(13)	0.0
Hospital category^†^	1553	(53)	667	(48)	9.7	1077	(50)	1077	(50)	0.0
*Region*										
Hokkaido/Tohoku	260	(8.8)	109	(7.8)	3.6	177	(08)	177	(8.1)	0.0
South Kanto	97	(3.3)	25	(1.8)	9.5	46	(02)	46	(2.1)	0.0
North Kanto/Koshin	272	(9.2)	107	(7.7)	5.6	182	(08)	182	(8.4)	0.0
Hokuriku	77	(03)	58	(4.2)	8.6	79	(04)	79	(3.6)	0.0
Tōkai	1,329	(45)	683	(49)	7.8	1,033	(47)	1,033	(47)	0.0
Kinki	628	(21)	258	(18)	7.0	427	(20)	427	(20)	0.0
Chugoku	23	(0.8)	9	(9.1)	39.1	15	(0.7)	15	(0.7)	0.0
Shikoku	247	(8.4)	127	(1.5)	32.1	194	(8.9)	194	(8.9)	0.0
Kyushu/Okinawa	20	(0.7)	21	(1.5)	8.0	22	(1.0)	22	(1.0)	0.0
*Year*										
2014	5	(0.2)	3	(0.2)	1.0	4	(0.2)	4	(0.2)	0.0
2015	235	(8.0)	99	(7.1)	3.3	157	(7.2)	157	(7.2)	0.0
2016	270	(9.1)	146	(10)	4.4	217	(10)	217	(10)	0.0
2017	227	(7.7)	126	(9.0)	4.8	185	(8.5)	185	(8.5)	0.0
2018	501	(17)	248	(18)	2.1	384	(18)	384	(18)	0.0
2019	423	(14)	217	(16)	3.4	328	(15)	328	(15)	0.0
2020	465	(16)	204	(15)	3.2	327	(15)	327	(15)	0.0
2021	442	(15)	182	(13)	5.6	299	(14)	299	(14)	0.0
2022	325	(11)	145	(10)	2.0	234	(11)	234	(11)	0.0
2023	60	(2.0)	27	(1.9)	0.7	40	(1.8)	40	(1.8)	0.0

The values represent numbers (percentages), unless stated otherwise

*ASD* Absolute standardized difference, *HER2* Human epidermal growth factor receptor 2

*An ASD ≥ 10% was considered to indicate a meaningful imbalance between the two groups

^†^Hospital category included academic hospitals and designated cancer care hospitals

Table 3 presents the incidences of the outcomes after overlap weighting. The adjusted incidence of composite cardiovascular disease in the denosumab and zoledronic acid groups was 118 and 152 per 10,000 person-years, respectively, with a difference of –34 cases per 10,000 person years. The crude incidence rates are presented in Table S2.

**Table 3:** Incidence of outcomes after overlap weighting

	Incidence (per 10000 person-years)		
	Denosumab	Zoledronic Acid	Difference
*Primary outcome*			
Composite cardiovascular disease*	118	152	− 34
*Secondary outcomes*			
Heart failure	65	92	− 27
Myocardial infarction	7.7	8.1	− 0.4
Stroke	51	55	− 4
Composite outcome of any fracture^†^	237	298	− 61
Hip fracture	31	43	− 12
Vertebral fracture	135	168	− 33
Non-vertebral fracture	114	142	− 28
All-cause mortality	471	610	− 139

*CI* Confidence interval

*Composite cardiovascular disease was defined as hospitalization for at least one of the following: stroke, acute myocardial infarction, or heart failure. Heart failure was defined as hospitalization for heart failure with initiation of the following medication (i.e., intravenous furosemide, carperitide, or tolvaptan) within 30 days of admission

^**†**^Fracture events were defined as those with a diagnosis of fracture accompanied by hospitalization. The composite outcome of any fracture was defined as a combination of hip, vertebral, and nonvertebral fractures. Non-vertebral fractures included fractures at any site except the vertebral fracture, such as fractures of the hip, pelvis, femur, leg, ankle, shoulder, forearm, and wrist

**Fig. 2:**
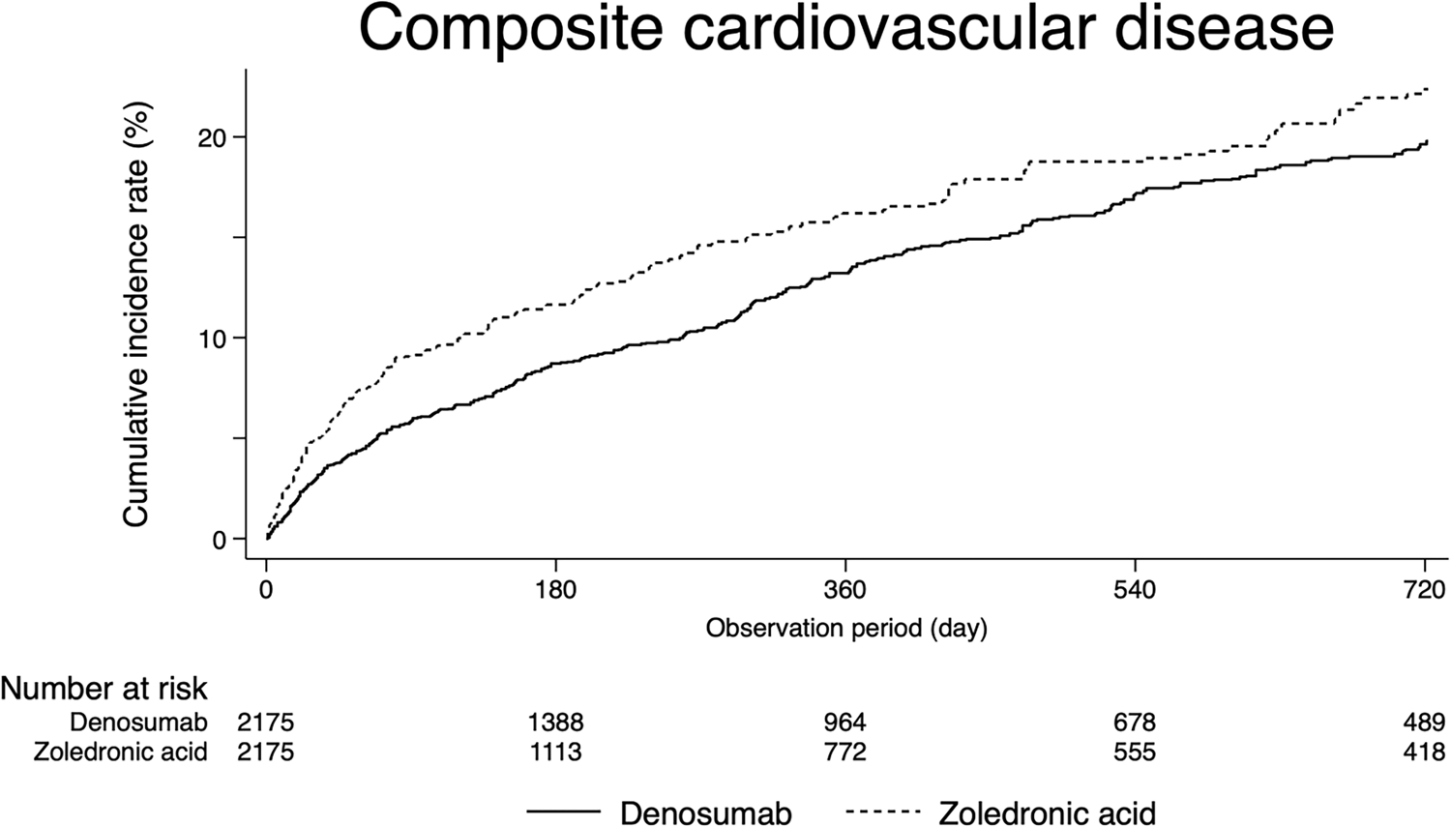
Cumulative probability for composite cardiovascular disease

Figure 2 shows the cumulative probability of composite cardiovascular disease. Composite cardiovascular disease occurred less frequently in the denosumab group than in the zoledronic acid group (log-rank test, *P* = 0.01).

**Fig. 3:**
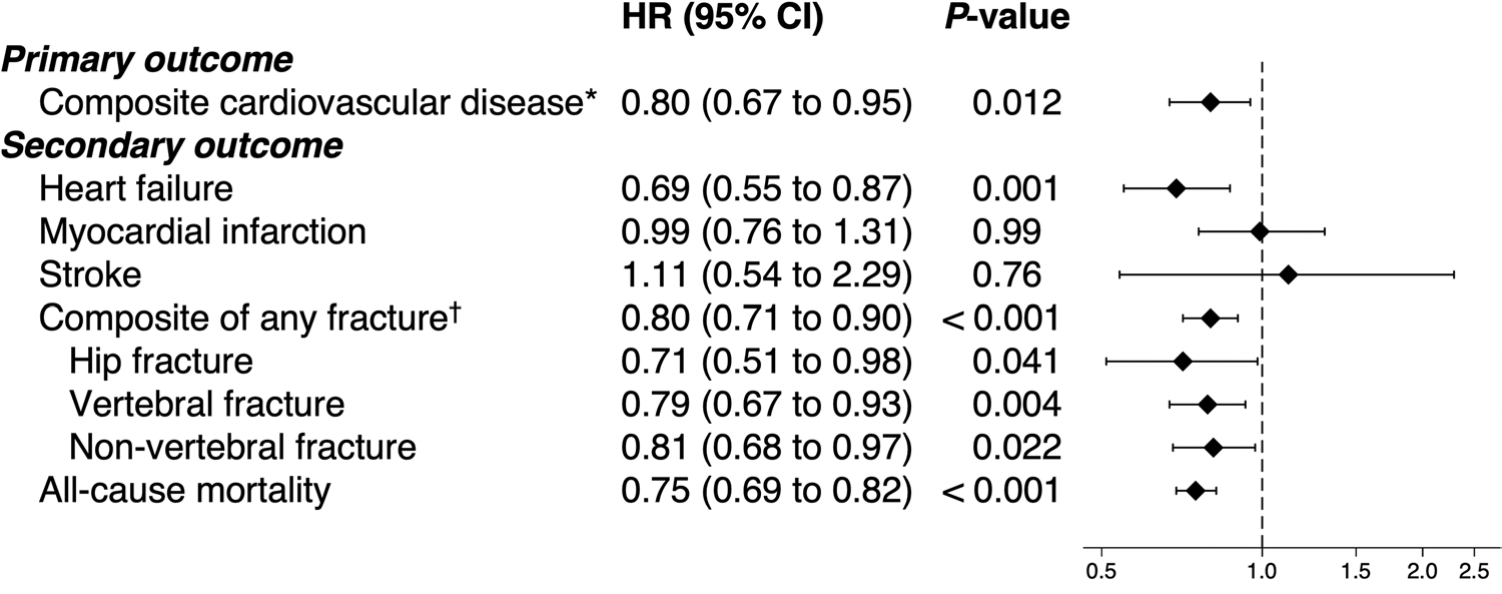
Adjusted hazard ratios and 95% CI for outcomes after overlap weighting. HR, hazard ratio; CI, confidence interval

Figure 3 shows the adjusted HRs for the outcomes after overlap weighting. The risk of incident composite cardiovascular disease was significantly lower in the denosumab group than in the zoledronic group [HR 0.80; 95% confidence interval (CI), 0.67 to 0.95]. For the secondary outcomes, the risk of incident heart failure was significantly lower in the denosumab group [HR 0.69 (95% CI, 0.55 to 0.87)] than in the zoledronic acid group, while the risk of myocardial infarction and stroke did not differ significantly between the two groups. Denosumab use was also associated with significantly lower risks of any fracture [HR 0.80 (95% CI, 0.71 to 0.90)] and all-cause mortality [HR 0.75 (95% CI, 0.69 to 0.82)]. This difference was similar for all fracture types, including hip, vertebral, and nonvertebral fractures. The crude results are presented in Supplementary Table S3.

Several sensitivity analyses revealed trends similar to those observed in the main analysis (Tables S4 and S5). The HR of the falsification analysis was 1.03 (0.48 to 2.20).

## Discussion

This large-scale retrospective analysis of real-world clinical data compared the risks of cardiovascular, fracture, and mortality events between denosumab and zoledronic acid administration in female patients with breast cancer bone metastases. Notably, denosumab was associated with a lower risk of hospitalization for heart failure after adjusting for several background factors. Additionally, compared to zoledronic acid, denosumab was associated with a lower fracture incidence and all-cause mortality.

A potential biological explanation may exist for the findings of the current study. Vascular calcification has been identified as a pathological factor in cardiovascular diseases, including atherosclerosis, hypertension, and coronary artery disease [30]. This mechanism involves inflammatory processes wherein cytokines and oxidative stress lead to endoplasmic reticulum stress, resulting in vascular smooth muscle cell calcification [30, 31]. The osteoprotegerin (OPG)–RANK–RANKL pathway has been implicated in cardiovascular pathology. Studies have found elevated levels of OPG, RANK, and RANKL in the myocardial tissues and circulation of patients with heart failure. RANKL increases matrix metalloproteinase activity in human fibroblasts, which affects left ventricular function [32]. Denosumab is a RANKL inhibitor that acts through the OPG–RANK–RANKL pathway [33] and may theoretically confer cardiovascular protective effects.

Studies have reported conflicting results regarding the effect of bisphosphonates on cardiovascular disease. One study suggested that bisphosphonates may inhibit the differentiation of vascular smooth muscle cells into osteoblast-like cells and regulate the OPG–RANK–RANKL pathway, potentially suppressing atherosclerosis and reducing the risk of cardiovascular disease [34]. However, another study suggested that bisphosphonates may induce inflammation, leading to instability or rupture of arterial plaques [35]. The current study showed that bisphosphonates were associated with a higher incidence of cardiovascular events compared with denosumab.

A previous randomized controlled trial comparing denosumab with zoledronic acid for the treatment of bone metastases in breast cancer was primarily designed to evaluate skeletal-related events and did not assess cardiovascular disease as a prespecified endpoint [8]. Due to the small number of events, no difference was observed in the cardiovascular events between the treatment groups. In contrast, the current study revealed that denosumab was associated with a lower risk of cardiovascular disease than zoledronic acid. These results are consistent with the findings of another meta-analysis; in 38,845 cancer-free adults aged ≥ 50 years, denosumab was associated with a lower risk of composite cardiovascular disease (relative risk, 0.82; 95% CI, 0.70 to 0.96), although the dosage greatly differs between bone metastasis and osteoporosis [11].

This study found that denosumab was associated with a significant reduction in all fracture types compared with zoledronic acid. This aligns with results from previous studies reporting an odds ratio of 0.86 (95% CI, 0.74 to 0.99) for fractures with denosumab [36] and a 39% reduction in fracture incidence in patients with breast cancer [37]. The observed reduction in hip fractures is particularly impactful, given their strong association with increased mortality (e.g., 5- to 8-fold within 3 months) [38] and functional decline [39] in elderly patients, with an elevated risk persisting for years [40].

In the current study, all-cause mortality was significantly lower in the denosumab group. This finding contrasts with previous randomized controlled trials that did not show a survival difference, presumably due to few cardiovascular events, strict eligibility criteria, and a young study population [41, 42]. The current study provides crucial real-world evidence of the overall survival benefit of denosumab. Because pathologic fracture would act as a critical mediator influencing overall survival, the fracture prevention effect of denosumab may have contributed to reduced mortality, particularly in this vulnerable age group where the fracture-related mortality risk is exceptionally high.

In Japan, the monthly cost of denosumab is approximately 293 USD, whereas that of zoledronic acid is approximately 79 USD per 3–4 weeks, based on the approximate 2025 exchange rate of 150 JPY per 1 USD. Clinicians should carefully balance these costs against the potential clinical benefits of denosumab, such as the reduction in cardiovascular events and fractures. The benefits of denosumab may outweigh its cost in older patients or high-risk patients with comorbidities, whereas zoledronic acid may remain a reasonable option for lower-risk patients.

### Clinical significance

Denosumab was associated with a lower risk of cardiovascular events, fractures, and all-cause mortality, highlighting important clinical implications for treatment selection in patients with breast cancer. Denosumab may be particularly beneficial in elderly patients with a history of cardiovascular disease or those at a high risk of fractures.

### Limitations

This study had some limitations. First, information on breast cancer subtypes was not available in the database. Because breast cancer subtypes influence both prognosis and the use of potentially cardiotoxic systemic therapies, the absence of this information may have resulted in residual confounding. Instead, we adjusted for the use of systemic therapies, including endocrine therapy, CDK4/6 inhibitors, and HER2-targeted therapies, which can be a proxy of subtypes. Second, we were unable to obtain data on the severity of renal impairment and important cardiovascular risk factors. Zoledronic acid is contraindicated in patients with severe renal impairment (creatinine clearance < 30 mL/min) [43]. Therefore, the denosumab group may have included patients with more severe renal impairment than the zoledronic acid group. Renal impairment is a well-established risk factor for cardiovascular disease [44], and the treatment effect of denosumab may have been underestimated. However, we consider the point estimates unlikely to be biased in the opposite direction. Additionally, although we did not adjust for cardiovascular risk factors (e.g., smoking history, alcohol consumption, and family history), we consider that these are unlikely to represent major unmeasured confounders because they exert little influence on the choice of exposure drugs. Third, because zoledronic acid, except for denosumab, is indicated for hypercalcemia of malignancy, patients who received zoledronic acid for hypercalcemia could have been included. However, although baseline laboratory data were unavailable to identify patients with hypercalcemia, we adjusted for the prior use of other treatments for hypercalcemia (i.e., intravenous bisphosphonates, calcitonin, and corticosteroids) as well as for diagnostic codes related to disorders of calcium metabolism, to minimize potential confounders. Fourth, detailed information on the location, number, and size of the bone metastases was not available. Instead, we adjusted for the history of pathological fractures and fracture-related surgeries, which may partially reflect the severity of bone metastasis. Fifth, the number of events for each component of the composite outcome (cardiovascular disease and fractures) was limited, and some components could not be analyzed using competing risk models, restricting interpretation of individual outcomes. Sixth, because recent clinical trials have demonstrated that 12-weekly zoledronic acid dosing is non-inferior to the conventional 3–4 weekly dosing [45–47], it has become the predominant practice in some countries. However, in Japan, zoledronic acid is still mainly administered every 3–4 weeks in the study period. Therefore, the effects observed in this study reflect the frequent dosing regimen and may not be necessarily generalizable to patients receiving zoledronic acid every 12 weeks. Seventh, because a large proportion of our study population comprised older patients the generalizability of the findings to younger patients with breast cancer may be limited. Finally, the median follow-up period was relatively short (< 1 year), which may not have been sufficient to fully capture long-term outcomes. Despite these inherent limitations of the observational study design, we confirmed the robustness of the results through several sensitivity analyses. Although the direction of estimates was consistent between cause-specific and Fine–Gray models, the subdistribution hazard ratios were closer to the null and lost statistical significance. Cause-specific models assess instantaneous risks among patients who remain alive and event-free, whereas Fine–Gray models evaluate the cumulative incidence function in the presence of competing risk of death [48]. Because mortality was higher in the zoledronic acid group, many patients died before they could experience cardiovascular disease or fracture, which lowered the cumulative incidence function in that group and reduced the between-group difference. As a result, the subdistribution hazard ratios were closer to the null compared with the cause-specific hazard ratios. The point estimates remained below unity in both models. This consistency indicates that the findings were aligned in direction across approaches. Furthermore, the falsification analyses indicated that the observed risk reductions were unlikely to stem from substantial residual confounding.

## Conclusion

Based on the analysis of a large real-world cohort that included a high proportion of older adults, denosumab was associated with a lower risk of cardiovascular events, fractures, and all-cause mortality than zoledronic acid in the treatment of bone metastases in patients with breast cancer.

## Supplementary Information

Below is the link to the electronic supplementary material.

Supplementary file1 (DOCX 91 kb)

## Data Availability

The data analyzed in this study are not publicly available because of contracts with hospitals providing data to the database. Further inquiries pertaining to the data can be directed to the corresponding author.

## Abbreviations

CI: Confidence interval
HR: Hazard ratio
ICD-10: International classification of diseases, 10th revision
OPG: Osteoprotegerin
RANK: Receptor activator of nuclear factor kappa-B
RANKL: Receptor activator of nuclear factor kappa-B ligand
