# Supplementary material for: Cardiovascular adverse events associated with denosumab versus zoledronic acid in patients with breast cancer: a propensity score overlap weighted analysis
Source: Breast Cancer Res Treat. 2025 Nov 25;215(1):16. doi: 10.1007/s10549-025-07852-x (PMC12647167; doi:10.1007/s10549-025-07852-x)
Supplement: Supplementary file 1 — Supplementary file1 (DOCX 91 kb) [file 10549_2025_7852_MOESM1_ESM.docx]

**Cardiovascular Adverse Events Associated with Denosumab Versus Zoledronic Acid in Patients with Breast Cancer: A Propensity Score Overlap Weighted Analysis**

Chikako Iwai, Takaaki Konishi, Atsushi Miyawaki, Akira Okada, Toshiaki Isogai, Taisuke Jo, Hideo Yasunaga

**Table S1** Definitions of ICD-10 Codes

**Table S2** Crude incidence of outcomes before overlap weighting

**Table S3** Crude hazard ratios for outcomes before overlap weighting

**Table S4** Adjusted subdistribution hazard ratios for outcomes after overlap weighting in the competing risk analysis

**Table S5** Adjusted hazard ratios for outcomes after overlap weighting in the full cohort (n = 5,504).

**Figure S1** Study design diagram

**Table S1** Definitions of ICD-10 Codes

| **Diseases** | **ICD-10 codes** |
| --- | --- |
| Breast cancer | C50 |
| ***Comorbidities*** |  |
| Angina | I20.0, I20.1, I20.8, I20.9 |
| Atrial fibrillation | I48 |
| Autoimmune disease | M05.0, M05.2, M05.3, M05.8, M05.9, M06, M30, M31, M32, M33, M34, M35, M36 |
|  | M35, M36 |
| Brain metastasis | C793 |
| Chronic kidney disease | N18 |
| Diabetes | E10.2–E10.5, E10.7, E11.2–E11.5, E11.7, E12.2–E12.5, E12.7, E13.2–E13.5, E13.7, |
|  | E14.2–E14.5, E14.7 |
|  | E10.0, E10.1, E10.6, E10.8, E10.9, E11.0, E11.1, E11.6, E11.8, E11.9, E12.0, E12.1, |
|  | E12.6, E12.8, E12.9, E13.0, E13.1, E13.6, E13.8, E13.9, E14.0, E14.1, E14.6, E14.8, E14.9 |
| Fracture |  |
| Hip fracture | S72.0, S72.1 |
| Vertebral fracture | S12, S22.0, S22.1, S32.0, T08 |
| Non-vertebral^*^ fracture^*^ | S32.1–S32.9, S42, S52, S62, S72.2–S72.9, S82, S92 |
| Heart failure | I11.0, I50 |
| Hypertension | I10, I12, I13, I15 |
| Myocardial infarction | I21.0, I21.1, I21.2, I21.3, I21.4, I21.9 |
| Osteoporosis | M81 |
| Stroke | I60–64 |

ICD-10, International Classification of Diseases, Tenth Revision

*Non-vertebral fractures included fractures at any site, except the hip and vertebrae, such as fractures of the pelvis, femur, leg, ankle, shoulder, forearm, and wrist.

**Table S2** Crude incidence of outcomes before overlap weighting

|  | **Incidence (per 10000 person-years)** | | |
| --- | --- | --- | --- |
|  | **Denosumab** | **Zoledronic Acid** | **Difference** |
| **Primary outcome** |  |  |  |
| Composite cardiovascular disease**^*^** | 106 | 162 | –56 |
| **Secondary outcomes** |  |  |  |
| Heart failure | 58 | 99 | –41 |
| Myocardial infarction | 7.0 | 7.7 | –0.7 |
| Stroke | 46 | 59 | –13 |
| Composite outcome of any fracture**^†^** | 214 | 312 | –98 |
| Hip fracture | 23 | 37 | –14 |
| Vertebral fracture | 122 | 179 | –57 |
| Non-vertebral fracture | 103 | 146 | –43 |
| All-cause mortality | 412 | 635 | –223 |

CI, confidence interval

*****Composite cardiovascular disease was defined as hospitalization for at least one of the following: stroke, acute myocardial infarction, or heart failure. Heart failure was defined as hospitalization for heart failure with initiation of the following medication (i.e., intravenous furosemide, carperitide, or tolvaptan) within 30 days of admission.

**†**Fracture events were defined as those with a diagnosis of fracture accompanied by hospitalization. The composite outcome of any fracture was defined as a combination of hip, vertebral, and nonvertebral fractures. Non-vertebral fractures included fractures at any site except the vertebral fracture, such as fractures of the hip, pelvis, femur, leg, ankle, shoulder, forearm, and wrist.

**Table S3** Crude hazard ratios for outcomes before overlap weighting

|  | **HR (95% CI)** | ***P-*value** |
| --- | --- | --- |
| **Primary outcome** |  |  |
| Composite cardiovascular disease^*^ | 0.81 (0.68 to 0.97) | 0.019 |
| **Secondary outcomes** |  |  |
| Heart failure | 0.71 (0.56 to 0.88) | 0.002 |
| Stroke | 1.02 (0.78 to 1.34) | 0.90 |
| Myocardial infarction | 1.10 (0.52 to 2.34) | 0.81 |
| Composite outcome of any fracture**^†^** | 0.81 (0.71 to 0.91) | 0.001 |
| Hip fracture | 0.73 (0.53 to 1.01) | 0.059 |
| Vertebral fracture | 0.78 (0.68 to 0.94) | 0.007 |
| Non-vertebral fracture | 0.72 (0.61 to 0.84) | < 0.001 |
| All-cause mortality | 0.75 (0.69 to 0.82) | < 0.001 |

HR, hazard ratio; CI, confidence interval

Median age was 73 (interquartile range, 66 to 80) years.

*****Composite cardiovascular disease was defined as hospitalization for at least one of the following: stroke, acute myocardial infarction, or heart failure. Heart failure was defined as hospitalization for heart failure with initiation of the following medication (i.e., intravenous furosemide, carperitide, or tolvaptan) within 30 days of admission.

**†**Fracture events were defined as those with a diagnosis of fracture accompanied by hospitalization. The composite outcome of any fracture was defined as a combination of hip, vertebral, and nonvertebral fractures. Non-vertebral fractures included fractures at any site, except the vertebral fracture, such as fractures of the hip, pelvis, femur, leg, ankle, shoulder, forearm, and wrist.

**Table S4** Adjusted subdistribution hazard ratios for outcomes after overlap weighting in the competing risk analysis

|  | **SHR** | **95% CI** | ***P-*value** |
| --- | --- | --- | --- |
| **Primary outcome** |  |  |  |
| Composite cardiovascular disease**^*^** | 0.89 | 0.75 to 1.06 | 0.19 |
| **Secondary outcomes** |  |  |  |
| Heart failure | 0.80 | 0.64 to 0.99 | 0.045 |
| Stroke | NA | NA | NA |
| Myocardial infarction | NA | NA | NA |
| Composite of any fractures**^†^** | 0.90 | 0.80 to 1.01 | 0.081 |
| Hip fracture | NA | NA | NA |
| Vertebral fracture | NA | NA | NA |
| Non-vertebral fracture | NA | NA | NA |

NA: Not available due to insufficient data for analysis.

CI, confidence interval

The results reflect the effect estimates under a competing risk framework, which accounts for the fact that death may preclude the occurrence of the outcome of interest.

*****Composite cardiovascular disease was defined as hospitalization for at least one of the following: stroke, acute myocardial infarction, or heart failure. Heart failure was defined as hospitalization for heart failure with initiation of the following medication (i.e., intravenous furosemide, carperitide, or tolvaptan) within 30 days of admission.

**†**Fracture events were defined as those with a diagnosis of fracture accompanied by hospitalization. The composite outcome of any fracture was defined as a combination of hip, vertebral, and nonvertebral fractures. Non-vertebral fractures included fractures at any site, except the vertebral fracture, such as fractures of the hip, pelvis, femur, leg, ankle, shoulder, forearm, and wrist.

**Table S5** Adjusted hazard ratios for outcomes after overlap weighting in the full cohort (*n* = 5,504)

|  | **HR (95% CI)** | ***P-*value** |
| --- | --- | --- |
| **Primary outcome** |  |  |
| Composite cardiovascular disease^*^ | 0.69 (0.68 to 0.93) | 0.005 |
| **Secondary outcomes** |  |  |
| Heart failure | 0.69 (0.57 to 0.86) | 0.001 |
| Stroke | 0.95 (0.74 to 1.23) | 0.71 |
| Myocardial infarction | 1.14 (0.68 to 2.11) | 0.68 |
| Composite outcome of any fracture**^†^** | 0.83 (0.74 to 0.93) | 0.002 |
| Hip fracture | 0.74 (0.55 to 1.01) | 0.058 |
| Vertebral fracture | 0.84 (0.73 to 0.98) | 0.025 |
| Non-vertebral fracture | 0.84 (0.71 to 0.99) | 0.033 |

HR, hazard ratio; CI, confidence interval

Median age was 73 (interquartile range, 66 to 80) years.

This full cohort included all patients regardless of the availability of mortality data. In other words, it encompassed all insurance cohorts, including the employee health insurance cohort, which consisted mainly of patients aged < 65 years; follow-up censored at the last observed data point or the end of the study.

*****Composite cardiovascular disease was defined as hospitalization for at least one of the following: stroke, acute myocardial infarction, or heart failure. Heart failure was defined as hospitalization for heart failure with initiation of the following medication (i.e., intravenous furosemide, carperitide, or tolvaptan) within 30 days of admission.

**†**Fracture events were defined as those with a diagnosis of fracture accompanied by hospitalization. The composite outcome of any fracture was defined as a combination of hip, vertebral, and nonvertebral fractures. Non-vertebral fractures included fractures at any site, except the vertebral fracture, such as fractures of the hip, pelvis, femur, leg, ankle, shoulder, forearm, and wrist.

**Figure S1** Study design diagram


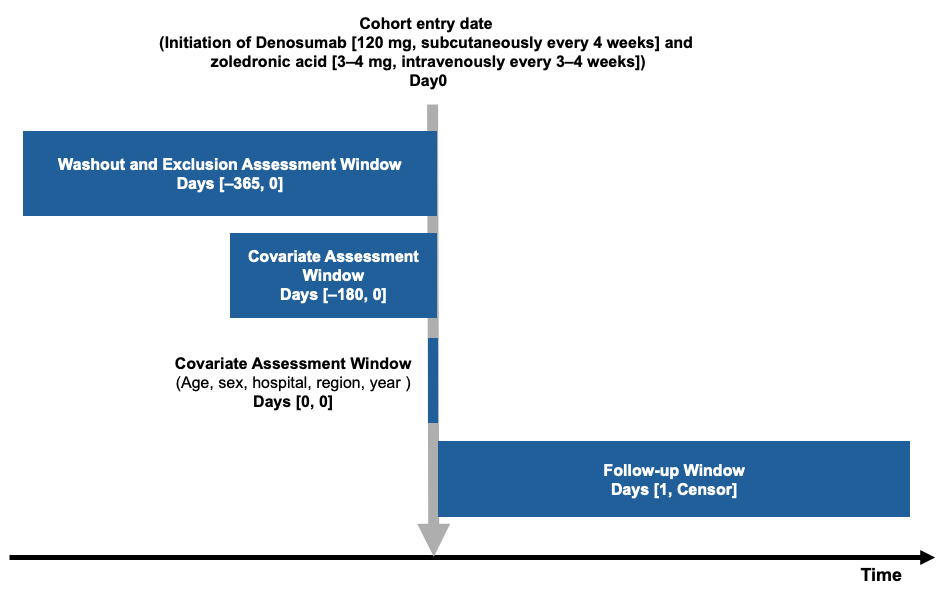


The pre-index washout and assessment period [days (–365, 0)] served to ensure no prior exposure to the index treatments, denosumab or zoledronic acid, and to identify patients who met the exclusion criteria. The exclusion criteria during this period included patients without mortality data, those who joined insurers within 1 year before the index date (i.e., those without a washout period), and those with prior intravenous zoledronic acid (5 mg per year) or subcutaneous denosumab (60 mg every 6 months) administration, as such administration was deemed to be for osteoporosis. Covariates were assessed based on data from day 0 (e.g., age, sex, hospital, region, and year) or within a window of days (–180, 0) (e.g., specific comorbidities, medications).
